# Cardioembolic but Not Other Stroke Subtypes Predict Mortality Independent of Stroke Severity at Presentation

**DOI:** 10.4061/2011/281496

**Published:** 2011-10-10

**Authors:** Latha Ganti Stead, Rachel M. Gilmore, M. Fernanda Bellolio, Anunaya Jain, Alejandro A. Rabinstein, Wyatt W. Decker, Dipti Agarwal, Robert D. Brown

**Affiliations:** ^1^Department of Emergency Medicine, Mayo Clinic, Rochester, MN 55905, USA; ^2^Division of Clinical Research, Department of Emergency Medicine, University of Florida, Gainesville, FL 32610, USA; ^3^Department of Neurosurgery, University of Rochester, Rochester, NY 14642, USA; ^4^Department of Neurology, Mayo Clinic, Rochester, MN 55902, USA

## Abstract

*Introduction*. Etiology of acute ischemic stroke (AIS) is known to significantly influence management, prognosis, and risk of recurrence. *Objective*. To determine if ischemic stroke subtype based on TOAST criteria influences mortality. *Methods*. We conducted an observational study of a consecutive cohort of patients presenting with AIS to a single tertiary academic center. *Results*. The study population consisted of 500 patients who resided in the local county or the surrounding nine-county area. No patients were lost to followup. Two hundred and sixty one (52.2%) were male, and the mean age at presentation was 73.7 years (standard deviation, SD = 14.3). Subtypes were as follows: large artery atherosclerosis 97 (19.4%), cardioembolic 144 (28.8%), small vessel disease 75 (15%), other causes 19 (3.8%), and unknown 165 (33%). One hundred and sixty patients died: 69 within the first 30 days, 27 within 31–90 days, 29 within 91–365 days, and 35 after 1 year. Low 90-, 180-, and 360-day survival was seen in cardioembolic strokes (67.1%, 65.5%, and 58.2%, resp.), followed for cryptogenic strokes (78.0%, 75.3%, and 71.1%). Interestingly, when looking into the cryptogenic category, those with insufficient information to assign a stroke subtype had the lowest survival estimate (57.7% at 90 days, 56.1% at 180 days, and 51.2% at 1 year). *Conclusion*. Cardioembolic ischemic stroke subtype determined by TOAST criteria predicts long-term mortality, even after adjusting for age and stroke severity.

## 1. Introduction

Etiology of acute ischemic stroke (AIS) is known to significantly influence management, prognosis, and risk of recurrence. Certain stroke subtypes are associated with higher stroke severity at the time of presentation, which may account for the higher mortality seen. In 1993 the TOAST (Trail of ORG 10172 in Acute Stroke Treatment) investigators described a classification of AIS based on etiology, which is now the most commonly used etiological classification [1]. Comparison of clinical characteristics, functional outcomes, and mortality rates for specific ischemic stroke mechanisms may allow clinicians to identify those patients who are at higher risk and to evaluate treatment strategies more definitely. 

We conducted an observational study of all patients who presented to the emergency department (ED) with AIS and determined if ischemic stroke subtype (ISS) influences mortality even after correcting for stroke severity on initial presentation.

## 2. Methods

This study was conducted at a tertiary care academic medical center, with an annual ED census of approximately 75,000 visits. The medical records of all patients with a discharge diagnosis of stroke or transient ischemic attack (TIA) or diagnoses which could be mistaken for stroke or TIA were screened (ICD-CM codes 433-437) between December 2001 and March 2004 to determine if the case met the criteria for diagnosis of ischemic stroke. This study was approved by the authors' institutional review board.

The medical records for all patients were reviewed, and details of clinical history, demographic information, risk factor profile, neurological examination, brain imaging studies, and other diagnostic studies were abstracted. Followup was updated for the final data analysis using the date of the last service or dismissal available from registration databases. In addition, dates and causes of death were ascertained from the State of Minnesota Electronic Death Certificate Data; autopsy reports were also reviewed where available. The National Institutes of Health Stroke Scale (NIHSS) was calculated by physicians (LGS, RMG) certified in the NIHSS, based on previously validated methods [2, 3]. The scoring was derived from documentation of the neurological examination performed by the neurologist on call at the time of ED presentation. Stroke subtype was assigned to every patient by two independent physicians based on review of clinical history, neurological examination, and diagnostic studies in accordance with criteria outlined in the TOAST study. The TOAST classification includes the following categories: (1) large artery (LAD), (2) cardioembolic (CE), (3) small vessel (SAD), (4) other determined cause, and (5) cryptogenic. 

Patients defined as having large-artery atherosclerosis had imaging showing greater than 50% stenosis or occlusion of a major brain artery or branch cortical artery supplying the region of brain affected. A cardiac source of stroke was assigned to patients with at least one of the following predisposing factors (1) prosthetic valve, (2) significant mitral stenosis, (3) atrial fibrillation or flutter, (4) left atrial or ventricular thrombus, (5) myocardial infarction <6 months prior to stroke, (6) dilated cardiomyopathy, (7) akinetic/hypokinetic left ventricular segment, (8) atrial myxoma, (9) infective endocarditis, (10) sick-sinus syndrome, and (11) congestive heart failure, in the absence of significant ipsilateral arterial stenosis. Patients with small vessel occlusion had a clinical history of a classical lacunar syndrome with either no evidence of infarction on neuroimaging or less than 1.5 cm infarct in the corresponding subcortical or brainstem region. Patients with evidence of stroke of other more unusual etiologies such as vasculitis or hypercoagulable states were classified as stroke of other determined cause. Patients who did not meet the criteria for any of the above categories were defined as having cryptogenic stroke. This category was comprised of 3 distinct groups of patients (a) those that had more than one cause identified, (b) no cause identified despite extensive investigations, and (c) insufficient information obtained to identify a cause.

## 3. Statistical Methods

The primary outcome variable was mortality within 1 year, as estimated using the Kaplan-Meier method. For patients who died within 1 year, the duration of followup was calculated from the date of ED presentation to the date of death. The duration of followup for all remaining patients was censored at the date of last followup if within 1 year or at 366 days. Associations with survival were evaluated using indicator variables in Cox proportional hazards models. The associations were evaluated with and without adjusting for sex, age, and NIHSS (ln (score + 0.5) of NIHSS which was used in the model) and were summarized by calculating risk ratios (RRs) and 95% confidence intervals (CIs). All calculated *P* values were two sided, and *P* values less than 0.05 were considered statistically significant. Statistical analyses were performed using the SAS software package (SAS Institute, Inc, Cary, NC).

## 4. Results

The initial study population consisted of all 681 consecutive patients who presented to the ED with acute ischemic stroke. Among these 681 patients, 19 denied research authorization and were therefore excluded from further study in accordance with Minnesota Statute 144.335. For purposes of obtaining recent and consistent followup, this sample was further limited to the 500 patients who resided in the local county or the surrounding nine-county area at the time of the ED visit. Hence, during the period of study 500 eligible patients were identified. No patients were lost to followup. 

Two hundred and sixty one (52.2%) were male, and the mean age at presentation was 73.7 years (standard deviation, SD = 14.3, range 18 to 101 years). Magnetic resonance imaging or computed tomography (CT) of the brain was carried out in all patients with 60% of patients having both imaging modalities. Echocardiography was performed in 65% of patients, 92% of which were transesophageal echocardiography. 85% had vascular imaging with 65% of these having CT angiogram or magnetic resonance angiogram.

All 500 patients were assigned a subtype, large artery atherosclerosis 97 (19.4%), cardioembolic 144 (28.8%), small vessel disease 75 (15%), other determined cause 19 (3.8%), and unknown 165 (33%). The unknown category was further subdivided into: more than one cause identified 37 (7.4%), no cause identified 63 (12.6%), and those with insufficient investigations performed to identify a cause 65 (13.0%). Detailed information of the demographic characteristics by ISS is presented in Table 1.

**Figure 1:**
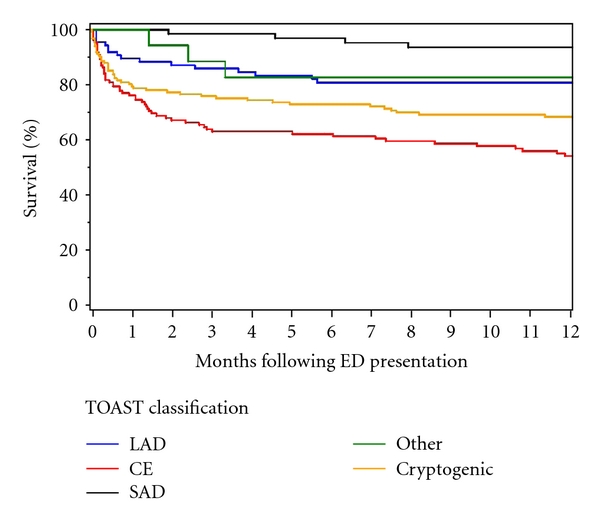
Survival estimates by TOAST classification.

One hundred and sixty patients died: 69 within the first 30 days, 27 within 31–90 days, 29 within 91–365 days, and 35 after 1 year. The estimated survival (±standard error) at 90 days, 180 days, and 1 year was 80.1% ± 1.8%, 77.5% ± 1.9%, and 73.5% ± 2.0%, respectively. Among the patients alive at last followup, the median followup was 1.8 years (interquartile range, 1.1–2.6 years). The lower 90-, 180-, and 360-day survival was seen in cardioembolic strokes (67.1%, 65.5%, and 58.2%, resp.), followed for cryptogenic strokes (78.0%, 75.3%, and 71.1%). Interestingly, when looking into the cryptogenic category, those with insufficient information to assign a stroke subtype had the lowest survival estimate (57.7% at 90 days, 56.1% at 180 days and 51.2% at 1 year). The survival estimates (±standard error) by TOAST category are presented in Table 2 and Figure 1. 

In order to calculate risk ratios (RR) for comparing the survival within the first year across the TOAST categories, we built 3 models to adjust for different factors. These models are summarized in Table 3. Using the SAD group as the reference, patients with LAD, CE, or cryptogenic strokes had significantly poorer survival (Model 1). This association was still observed after adjusting for age and gender (Model 2). However, after adjusting for age and NIHSS (Model 3), only the difference between CE and SAD attained statistical significance, with a RR 3.4 (95% CI 1.2–9.6). 

Most deaths (60%) in our cohort were attributable to the acute stroke itself, confirming the results of others [4]. Other causes were respiratory distress/pneumonia (15%), cardiovascular (myocardial infarction and congestive heart failure) (7%), and renal failure (7%).

We are unable to comment on whether there was a difference in tPA treatment or revascularization between subtypes as we did not have patient-specific data on which patients in this dataset as to who received t-PA by subtype. Overall, the number of patients who received t-PA during the study period was small and thus would likely have little effect on outcomes by subtype.

## 5. Discussion

Cardioembolic stroke is known to have the worst prognosis amongst ischemic stroke subtypes, and this has been reported in the literature [4, 5]. However, in contrast to the current study, these studies have failed to show CE stroke as an *independent* predictor of mortality. Bang et al. showed that ischemic stroke subtype was a significant predictor of recurrent stroke after adjusting for potential confounders but did not find that it was an independent predictor of poor prognosis [6]. Sprigg et al. reported in their evaluation of the TAIST (Tinzaparin in Acute Ischemic Stroke Trial) that patients with small vessel occlusion had better outcome as compared to patients with large artery atherosclerosis or cardioembolic stroke [7]. Internationally there seems to be a similar trend. Recently Winter et al. reported from Marburg that patients with cardioembolic stroke had more severe clinical deficits on presentation, and a worse outcome, than the other stroke subtypes [8]. A community-based study from Brazil also reported the highest case fatality rate in strokes of undetermined etiology followed by cardioembolic stroke and large vessel atherothrombosis [9]. Lavados and colleagues from Chile reported a similar higher mortality for cardioembolic stroke when compared to small vessel disease [10]. 

We have demonstrated an association between CE stroke and increased mortality, independent of age, gender, and NIHSS. It would appear likely that cardiac and other comorbidities most likely explain this finding. Cardiac conditions that predispose to stroke (such as extensive acute myocardial infarction, chronic myocardial injury with left ventricular aneurysm formation, valvular and nonvalvular atrial fibrillation) are themselves associated with increased mortality. It is not unexpected that such patients would carry a particularly poor prognosis after acute ischemic stroke. Similarly, conditions such as diabetes that may underlie such heart diseases may also increase risk for poor outcome following stroke.

The findings of this study clearly identify patients presenting with CE stroke as a particularly high risk group. How best to optimize therapy and ameliorate that risk for these patients, however, is uncertain. For example, whether stroke subtype may influence efficacy of established therapies such as thrombolytic therapy is unknown. It is probably reasonable to assume that embolic material in CE stroke is predominantly thrombus laden (rather than composed of plaque debris, as may pertain in aortic and carotid artery disease). If this assumption is indeed correct, then one could hypothesize that patients with CE stroke may be a subgroup that ought to derive greater benefit from timely administration of thrombolysis and may conceivably benefit from thrombolysis over an extended time window beyond the conventional three hours from symptom onset. Greater vigilance in respect of blood pressure control during the acute phase of stroke may be warranted. Heightened awareness of poor prognosis among these patients may also lead to more aggressive treatment of coexisting cardiac disorders. 

## 6. Conclusion

Cardioembolic ischemic stroke subtype determined by TOAST criteria predicts long-term mortality, even after adjusting for age and stroke severity. Further studies to define the precise nature of this increased risk will be required to guide the development of strategies which may improve outcome in this setting. 

## Figures and Tables

**Table 1:** Summary of patient characteristics by TOAST classification.

	Large vessel (*N* = 97)	Cardioembolic (*N* = 144)	Small vessel (*N* = 75)	Other (*N* = 19)	Multiple causes (*N* = 37)	No cause identified (*N* = 63)	Insufficient info (*N* = 65)
Male gender	67 (69.1%)	66 (45.8%)	39 (52%)	10 (52.6%)	21 (56.8%)	32 (50.8%)	26 (40%)
Age							
Mean (SD)	70.7 (12.22)	78.4 (11.81)	72.6 (13.50)	50.7 (19.71)	73.8 (9.90)	66.6 (16.78)	82.3 (8.32)
Range	39–91	41–101	44–94	18–82	39–90	25–89	59–98
NIHSS							
Mean (SD)	7.0 (6.40)	10.1 (9.06)	3.8 (2.71)	5.7 (5.77)	8.1 (8.92)	6.3 (7.14)	13.4 (11.26)
Median	4.0	7.0	3.0	5.0	4.0	4.0	9.5
*Q*1, *Q*3	3.0, 10.0	3.0, 15.0	2.0, 5.0	1.0, 8.0	2.0, 8.0	1.0, 8.0	4.0, 20.0
Prior MI	14 (14.4%)	16 (11.1%)	11 (14.7%)	1 (5.3%)	13 (35.1%)	8 (12.7%)	9 (13.8%)
CHF	7 (7.2%)	46 (31.9%)	6 (8%)	0 (0%)	12 (32.4%)	2 (3.2%)	10 (15.4%)
Coronary artery disease	25 (25.8%)	40 (27.8%)	19 (25.3%)	3 (15.8%)	18 (48.6%)	13 (20.6%)	16 (24.6%)
Atrial fibrillation	8 (8.2%)	89 (61.8%)	3 (4%)	2 (10.5%)	15 (40.5%)	3 (4.8%)	6 (9.2%)
Prior stroke	22 (22.7%)	35 (24.3%)	13 (17.3%)	2 (10.5%)	11 (29.7%)	15 (23.8%)	19 (29.2%)
Prior TIA	19 (19.6%)	20 (13.9%)	14 (18.7%)	0 (0%)	8 (21.6%)	9 (14.3%)	12 (18.5%)
HTN	75 (77.3%)	110 (76.4%)	61 (81.3%)	8 (42.1%)	32 (86.5%)	48 (76.2%)	51 (78.5%)
Hyperlipidemia	58 (59.8%)	56 (38.9%)	43 (57.3%)	3 (15.8%)	18 (48.6%)	31 (49.2%)	23 (35.4%)
Diabetes	28 (%)	35 (%)	30 (%)	2 (%)	9 (%)	18 (%)	13 (%)
Smoker							
Never	32 (33%)	79 (54.9%)	44 (58.7%)	10 (52.6%)	20 (54.1%)	23 (36.5%)	30 (46.2%)
Active	24 (24.7%)	14 (9.7%)	13 (17.3%)	4 (21.1%)	2 (5.4%)	16 (25.4%)	6 (9.2%)
Former	40 (41.2%)	44 (30.6%)	17 (22.7%)	5 (26.3%)	12 (32.4%)	23 (36.5%)	25 (38.5%)

**Table 2:** Survival estimates within the first year.

TOAST	*N*	Events	Survival at 90 days (SE)	Survival at 180 days (SE)	Survival at 360 days (SE)
Large artery	97	16	87.1% (0.04)	82.4% (0.04)	82.4% (0.04)
Cardioembolic	144	57	67.1% (0.04)	65.5% (0.04)	58.2% (0.04)
Small vessels	75	4	98.6% (0.01)	97.1% (0.02)	94.2% (0.03)
Other	19	3	89.5% (0.07)	84.2% (0.08)	84.2% (0.08)
Cryptogenic	165	45	78.0% (0.03)	75.3% (0.03)	71.1% (0.04)
More than one cause	37	7	85.5% (0.06)	82.3% (0.07)	79.2% (0.07)
No cause	63	7	94.8% (0.03)	91.2% (0.04)	87.4% (0.05)
Insufficient info	65	31	57.7% (0.06)	56.1% (0.06)	51.2% (0.06)

**Table 3:** Risk ratios of mortality.

Factors	Model 1		Model 2		Model 3	
included	RR (95% CI)		RR (95% CI)		RR (95% CI)	

		*P* value		*P* value		*P* value

TOAST						
LAD	3.5 (1.2–10.4)	*P* = 0.027	4.0 (1.3–11.9)	*P* = 0.014	2.1 (0.7–6.4)	*P* = 0.18
CE	9.4 (3.4–25.9)	*P* < 0.001	7.8 (2.8–21.4)	*P* < 0.001	3.4 (1.2–9.6)	*P* = 0.020
SAD	Reference		Reference		Reference	Reference
Other	3.1 (0.7–13.9)	*P* = 0.14	9.0 (2.0–41.3)	*P* = 0.005	4.7 (1.0–21.6)	*P* = 0.05
Cryptogenic	18.9 (2.2–17.1)	*P* < 0.001	5.9 (2.1–16.3)	*P* < 0.001	2.5 (0.9–7.0)	*P* = 0.094
Female	n/a		0.9 (0.6–1.3)	*P* = 0.48	n/a	
Age*	n/a		1.8 (1.5–2.2)	*P* < 0.001	1.6 (1.3–1.9)	*P* < 0.001
Ln(NIHSS)**	n/a		n/a		3.6 (2.8–4.7)	*P* < 0.001

*Risk ratio per 10 year increase in age.

**Risk per a doubling in NIHSS score.
